# Yes-associated protein (YAP) in pancreatic cancer: at the epicenter of a targetable signaling network associated with patient survival

**DOI:** 10.1038/s41392-017-0005-2

**Published:** 2018-04-20

**Authors:** Enrique Rozengurt, James Sinnett-Smith, Guido Eibl

**Affiliations:** 1 Department of Medicine, David Geffen School of Medicine, University of California, Los Angeles, CA 9005 USA; 2 CURE: Digestive Diseases Research Center, University of California, Los Angeles, CA 9005 USA; 3 Molecular Biology Institute, University of California, Los Angeles, CA 9005 USA; 4 Department of Surgery, David Geffen School of Medicine, University of California, Los Angeles, CA 9005 USA

## Abstract

Pancreatic ductal adenocarcinoma (PDAC) is generally a fatal disease with no efficacious treatment modalities. Elucidation of signaling mechanisms that will lead to the identification of novel targets for therapy and chemoprevention is urgently needed. Here, we review the role of Yes-associated protein (YAP) and WW-domain-containing Transcriptional co-Activator with a PDZ-binding motif (TAZ) in the development of PDAC. These oncogenic proteins are at the center of a signaling network that involves multiple upstream signals and downstream YAP-regulated genes. We also discuss the clinical significance of the YAP signaling network in PDAC using a recently published interactive open-access database (www.proteinatlas.org/pathology) that allows genome-wide exploration of the impact of individual proteins on survival outcomes. Multiple YAP/TEAD-regulated genes, including *AJUBA*, *ANLN*, *AREG*, *ARHGAP29*, *AURKA*, *BUB1*, *CCND1*, *CDK6, CXCL5*, *EDN2*, *DKK1*, *FOSL1,FOXM1*, *HBEGF*, *IGFBP2*, *JAG1*, *NOTCH2*, *RHAMM*, *RRM2*, *SERP1*, and *ZWILCH*, are associated with unfavorable survival of PDAC patients. Similarly, components of AP-1 that synergize with YAP (*FOSL1*), growth factors (TGFα, EPEG, and HBEGF), a specific integrin (*ITGA2*), heptahelical receptors (*P2Y*_*2*_*R*, *GPR87*) and an inhibitor of the Hippo pathway (*MUC1*), all of which stimulate YAP activity, are associated with unfavorable survival of PDAC patients. By contrast, YAP inhibitory pathways (STRAD/LKB-1/AMPK, PKA/LATS, and TSC/mTORC1) indicate a favorable prognosis. These associations emphasize that the YAP signaling network correlates with poor survival of pancreatic cancer patients. We conclude that the YAP pathway is a major determinant of clinical aggressiveness in PDAC patients and a target for therapeutic and preventive strategies in this disease.

## Pancreatic cancer: Signaling network linked to patient survival

Yes-associated protein (YAP) signaling contributes to pancreatic cancer progression and is associated with poor patient survival. Previous studies have shown that YAP activates genes involved in cell proliferation to incite tumor growth and metastasis. Enrique Rozengurt and colleagues at University of California Los Angeles review the latest knowledge on YAP signaling and used the open access database The Human Protein Atlas to analyze the gene expression profile and prognosis of 176 patients with pancreatic ductal adenocarcinoma. Activation of upstream or downstream elements of the YAP signaling pathway correlated with shorter survival in patients. Conversely, the activation of signaling pathways that oppose YAP signaling were associated with a more favorable prognosis. These findings highlight YAP signaling pathway components as both prognostic markers and potential targets for developing much needed therapeutic and preventative strategies.

## Introduction

Pancreatic cancer, of which pancreatic ductal adenocarcinoma (PDAC) represents the most common histological subtype, is one of the most lethal human diseases, with overall 5-year survival rates of 7% and a median survival period of 4–6 months^1^. The incidence of this disease in the United States is estimated to increase to 53,670 new cases in 2017. Indeed, deaths due to PDAC are projected to increase dramatically, making the disease the second leading cause of cancer-related deaths in the USA before 2030^2^. Novel targets and agents for therapy and chemoprevention are urgently needed and will most likely arise from a more detailed understanding of the signaling mechanisms that stimulate the promotion and progression of sub-malignant (initiated) cells into pancreatic cancer cells and from the identification of modifiable risk factors for PDAC. Identification of the molecular mechanisms of PDAC promotion and drug resistance will clearly guide the discovery of novel targets, prognostic markers, agents for therapy and prevention and will identify effective signature markers for use in specific and personalized therapeutic procedures.

PDAC arises from the evolution of precursor lesions, the most common of which are pancreatic intraepithelial neoplasias (PanINs). Progression from these noninvasive ductal lesions to in situ carcinomas and invasive cancers is associated with the accumulation of genetic alterations, including activation of mutations in the *KRAS* oncogene, which are widely accepted to represent an initiating event^3^. Accordingly, exome sequencing has established *KRAS* to be the most frequently mutated gene in PDAC (~95%)^4, 5^. The majority of all missense *KRAS* mutations in PDAC occur at position G12, with a G12D single amino acid substitution being the most prevalent. These mutations prevent interactions between KRAS-GTP and KRAS GTPase-activating proteins (GAPs), thus leading to prolonged activation of KRAS and thereby to the activation of downstream signaling effectors, the best characterized of which are the RAF/MEK/ERK and PI3K/AKT/mTOR pathways^6, 7^. Genetically engineered mouse models that recapitulate many features of the human disease have defined a critical role for *Kras*^G12D^ in the initiation and maintenance of PDAC^3, 8, 9^. The progression of pancreatic carcinogenesis requires stimulation of a set of signaling pathways that lead to sustained cell proliferation^4^. Although, a *KRAS* mutation is an early and necessary event in the development of PDAC, it is not sufficient to promote the complete carcinogenic process. Activation of other pathways by additional mutations, including mutations in tumor suppressor genes, such as *TP53*, one of the most frequently mutated genes in human cancer, *CDKN2A*, the gene encoding p16 and p14, and *SMAD4* and/or environmental stimuli (obesity, type 2 diabetes mellitus) are required for the promotion of invasive PDAC. This article highlights a striking association between a signal transduction network and the overall survival of patients with PDAC.

## The hippo/yap/taz pathway and pdac

The transcriptional co-activators yes-associated protein (YAP)^10^ and its paralog WW-domain-containing Transcriptional co-Activator with a PDZ-binding motif (TAZ)^11^ are attracting intense attention as fundamental points of convergence and intersection of many signal transduction pathways that are implicated in the regulation of development, metabolism, organ-size, positional sensing, tissue regeneration and tumorigenesis^12–14^. Indeed, multiple products of the YAP/TEAD-regulated gene network have a major impact on these important processes, and YAP and TAZ are increasingly recognized as potent oncogenes in many cancer types^15^, especially in PDAC^16–19^. After a succinct overview of the YAP/TAZ network in PDAC, the basic tenet of this article will be to emphasize the association between the expression of each element of the network and patient overall survival. Therefore, this review integrates signal transduction and patient survival, and thus differs in focus from many recent excellent reviews on the Hippo/YAP/TAZ pathway that are available in the literature^12–15, 20^.

**Fig. 1:**
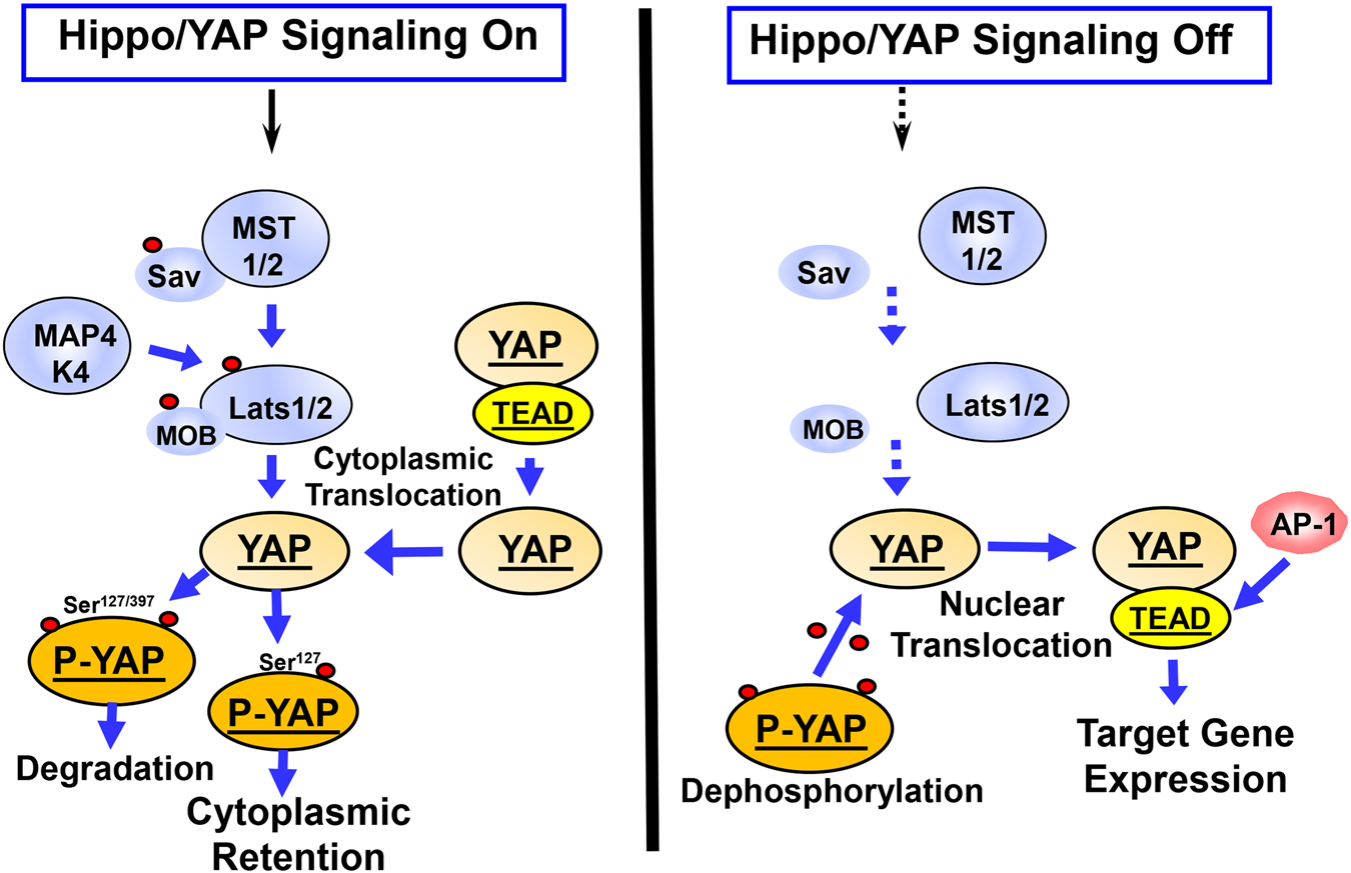
Hippo signaling phosphorylates YAP and regulates its nuclear/cytoplasmic distribution. When Hippo signaling is active (e.g., in response to cell density, polarity signals, or mechanical cues) the Mst1/2 kinases, in complex with Sav1, phosphorylate and activate Lats1/2 in complex with its regulatory protein MOB1/2. In addition to Mst1/2, MAP4Ks act as alternative kinases that phosphorylate Lats1/2. In turn, Lats1/2 phosphorylates YAP on highly conserved residues (in red) located within a consensus sequence that is phosphorylated by Lats1/2 (**H**X**R**XX**S**). The phosphorylation of YAP at Ser-127 promotes its cytoplasmic retention, whereas phosphorylation at Ser-397 induces degradation. When the Hippo pathway is off, YAP is dephosphorylated and translocated into the nucleus where YAP binds and activates the TEAD transcription factors and stimulates the expression of multiple genes. Additional details are provided in the text

It is widely recognized that a major factor in the regulation of YAP/TAZ activity is the Hippo pathway, which was originally identified in Drosophila^12^. Canonical Hippo signals are transduced through a serine/threonine kinase cascade, wherein Mst1/2 kinases, in complex with Sav1, phosphorylate and activate Lats1/2 in complex with its regulatory protein MOB1/2 (Fig. 1). In addition to Mst1/2, Hppy/MAP4Ks were identified as alternative kinases that phosphorylate Lats1/2^21^. In turn, Lats1/2 phosphorylates YAP and TAZ, two major downstream effectors of the Hippo pathway and novel sensors of the mevalonate and glycolytic pathways^13, 22, 23^. Structurally, YAP and TAZ share nearly half of their overall amino acid sequences, and have very similar topologies and highly conserved residues that are located within a consensus sequence that is phosphorylated by Lats1/2 (**H**X**R**XX**S**). The phosphorylation of YAP by Lats1/2 at Ser-127 and Ser-397 (and equivalent residues in TAZ) restricts its cellular localization to the cytoplasm and reduces the protein’s stability (Fig. 1). When the Hippo pathway is not functional, YAP and TAZ are dephosphorylated and translocated to the nucleus where they bind to and activate a number of transcription factors, primarily the TEA-domain DNA-binding transcription factors (TEAD 1–4). In this manner, nuclear YAP and TAZ promote the expression of multiple genes (Fig. 1). Accordingly, YAP and TAZ display a degree of functional redundancy^15, 20^ but also differ in a number of ways. For example, YAP negatively regulates TAZ, while TAZ expression levels do not modulate YAP levels^24^. TAZ is more unstable than YAP, thus these oncogenic proteins often are differentially expressed in different cancer cell types^25^. Therefore, activation of the tumor suppressor Hippo pathway in response to multiple environmental cues, including cell/cell contacts, cell polarity and mechanical tension, potently inhibits the transcriptional co-activator activity of YAP and TAZ and leads to the degradation of TAZ^12, 15, 26–28^.

**Fig. 2:**
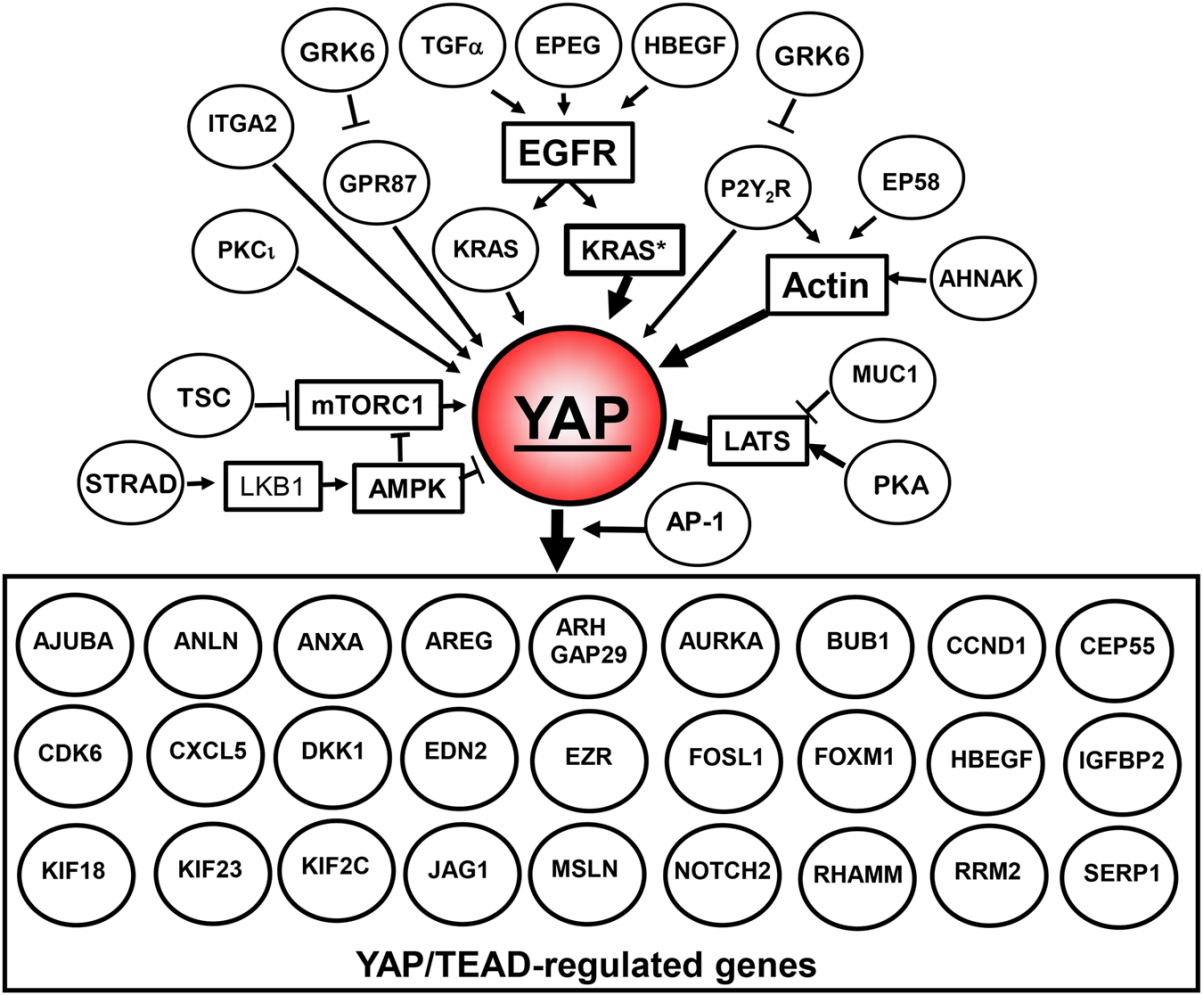
YAP is at the epicenter of a signaling network. Growth factors (TGFα, EPEG, and HBEGF) induce EGFR signaling leading to KRAS activation, which in turn, stimulates YAP activation and increased expression. Other tyrosine kinase receptors, a specific integrin (*ITGA2*), multiple GPCRs, an inhibitor of Hippo pathway (*MUC1*) and actin polymerization via multiple pathways also stimulate YAP activity. Activation of YAP stimulates its coupling with TEAD, thereby promoting the expression of multiple YAP/TEAD-regulated genes that were identified in screens in different cell types. Reference to the study or studies connecting each gene to YAP/TAZ, as well as further details concerning the network are in the text

By contrast, multitude upstream pathways positively control YAP/TAZ transcriptional co-activator activity. These include signals mediated by ligand-activated G protein-coupled receptors (GPCRs), tyrosine kinase receptors, integrins and mechanical cues (Fig. 2). A variety of signaling pathways that are activated by these receptors, including PI3K, mTOR, PKD, and Rho/actin cytoskeleton, feed into the YAP/TAZ pathway^12, 15, 26–28^. For example, a recent study with human PDAC cells demonstrated that crosstalk between insulin/IGF-1 receptor and GPCR systems^29, 30^ regulates YAP localization, phosphorylation, and transcriptional co-activator activity through PI3K and PKD^31^. Accordingly, recent studies with other cell types demonstrated that PI3K inhibits the activity of the Hippo pathway^32, 33^, thereby promoting YAP activity. Other studies have demonstrated that PKD, a key node downstream of GPCRs^34, 35^, stimulates the nuclear localization of YAP and the activation of YAP/TEAD-regulated gene expression, most likely by stabilizing the actin cytoskeleton^28^. Thus, extracellular stimuli can control YAP/TAZ activity via inhibition of the Hippo pathway and/or stabilization of the actin cytoskeleton, thereby regulating a complex program of gene expression (Fig. 2). A recent report indicated that in addition to YAP and TAZ, the TEAD transcription factors also shuttle between the nucleus and the cytoplasm and that environmental stress promotes the cytoplasmic translocation of TEAD via p38 MAPK in a Hippo-independent manner, thus identifying an additional level of regulation^36^.

The YAP/TAZ pathway assumes an added importance in PDAC because YAP is also a key downstream target of KRAS signaling^16^ that is required for acinar-to-ductal metaplasia (ADM) and PanIN progression into PDAC in genetically engineered mouse models^16, 17^. YAP is also a major mediator of pro-oncogenic mutant p53, resistance to RAF/MEK inhibitors and chemotherapy in PDAC^37, 38^, and TAZ supports development of pancreatic cancer^39^. Conversely, mutants that render p53 as a super suppressor of pancreatic cancer act via inactivation of Yap^40^. It is of interest that pancreas-specific deletion of *Yap* did not affect normal pancreatic development and endocrine functions, but blocked the progression of *Kras*^G12D^ –induced evolution of PanINs to overt PDAC^16^.

In addition to the rapid regulation of YAP activity via phosphorylation and localization, additional pathways and epigenetic events regulate the level of YAP/TAZ protein expression. In this regard, the RAS pathway promotes YAP1 stability independent of the Hippo pathway through down regulation of the ubiquitin ligase complex substrate recognition factors SOCS5/6, thereby increasing YAP stability^41^. A recent study demonstrated that eIF5A (eukaryotic translation initiation factor 5A), which is up-regulated by KRAS in PDAC and in KC mice (i.e., mice harboring a *Kras* ^G12D^), increases the tyrosine kinase PEAK1. In turn, the eIF5A/PEAK1 axis enhances the expression of YAP^42^. It is also relevant that YAP and mTORC1 form an amplification loop that enhances the expression of YAP protein. Specifically, YAP stimulates mTORC1 via down regulation of phosphatase and tensin homolog (PTEN) and increases amino acid (leucine) transport^43^. In turn, the activation of mTORC1 leads to the accumulation of YAP through impaired autophagy^44^. The positive feedback loop between YAP and mTORC1 increases YAP activity and expression.

Recent studies have demonstrated that amplification and overexpression of YAP could substitute for mutant *Kras* in murine cancers^19, 45^. These findings raise the important notion that YAP not only acts downstream of Kras but also that hyper-activation and expression of YAP can circumvent the need for *Kras* mutant expression in PDAC^3, 19^. An important feature of PDAC is the recruitment of immune-suppressive leukocytes, including myeloid-derived suppressor cells and tumor-associated macrophages, that contribute to immune evasion^3^. Recently, YAP has been shown to play a critical role in promoting an immunosuppressive microenvironment via the production of multiple cytokines in PDAC^46^ and in other cancers^47^. Thus, there is substantial evidence from preclinical studies indicating that YAP/TAZ is at the epicenter of a signaling network that is of crucial importance in the development of PDAC. Our next objective is to focus on the importance of the YAP/TAZ pathway in human PDAC.

## Clinical significance of yap: a prognostic marker in pdac

To assess the significance of the YAP pathway in human PDAC, we will discuss the importance of YAP as a prognostic marker of survival in patients with PDAC. A number of studies have indicated that YAP and TAZ are overactive in tumor samples from PDAC patients, as judged by their expression and or localization^18, 19, 39^. Furthermore, a recent report identified YAP expression as an independent prognostic marker for the overall survival of PDAC patients and its association with liver metastasis^48^. If YAP plays a critical role in the survival of PDAC patients, it is plausible that upstream and downstream elements of the network, as well as regulators of YAP activity that have been identified in a variety of cells, are also likely to be associated with survival of patients with PDAC, and can serve as prognostic markers. Given its important translational implications, we explored this proposition using a recently published interactive open-access database (www.proteinatlas.org/pathology) to perform correlation analyses based on mRNA expression levels of genes of the YAP pathway in PDAC tissue and the clinical outcome (survival) of the patients^49^. The data in the Pathology Atlas is based on the integration of publicly available data from The Cancer Genome Atlas and data generated within the framework of the Human Protein Atlas and analyzed transcriptomics and survival in 176 PDAC patients. The results are presented in the form of Kaplan–Meier plots and only differences in survival with a high statistical significance (*p* < 0.001) are taken into consideration. We also performed additional searches of the literature for studies using different patient cohorts that validate the conclusions drawn from the Pathology Atlas.

**Fig. 3:**
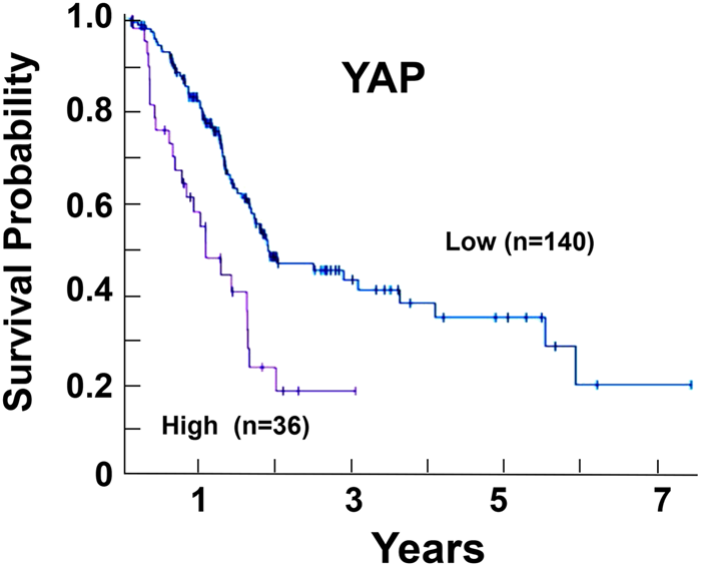
Kaplan–Meier plots for YAP expression in PDAC. The image was reproduced from the Human Protein Atlas (version 17) available at www.proteinatlas.org. The link is: http://www.proteinatlas.org/ENSG00000137693YAP1/pathology/tissue/pancreatic±cancer.

As expected, increased expression of YAP is associated significantly with an unfavorable prognosis (survival) in PDAC patients who were included in the Pathology Atlas^49^. Antibody staining, which is also included in the Pathology Atlas, is consistent with the mRNA expression data and is in agreement with other studies^39, 48^, localizes YAP/TAZ to the cancer cells. As illustrated in the Kaplan–Meier plot in Fig. 3, none of the patients of the population with higher levels of YAP mRNA expression (*n* = 36) survived for 5 years, although 32% of the population (*n* = 140) with lower levels of YAP mRNA survived for 5 years or more. In agreement with a recent report using a different cohort of patients^48^, an increase in the expression of YAP is an unfavorable prognostic marker for survival in patients with PDAC.

## Molecules downstream and upstream of yap are unfavorable prognostic markers in pdac

**Fig. 4:**
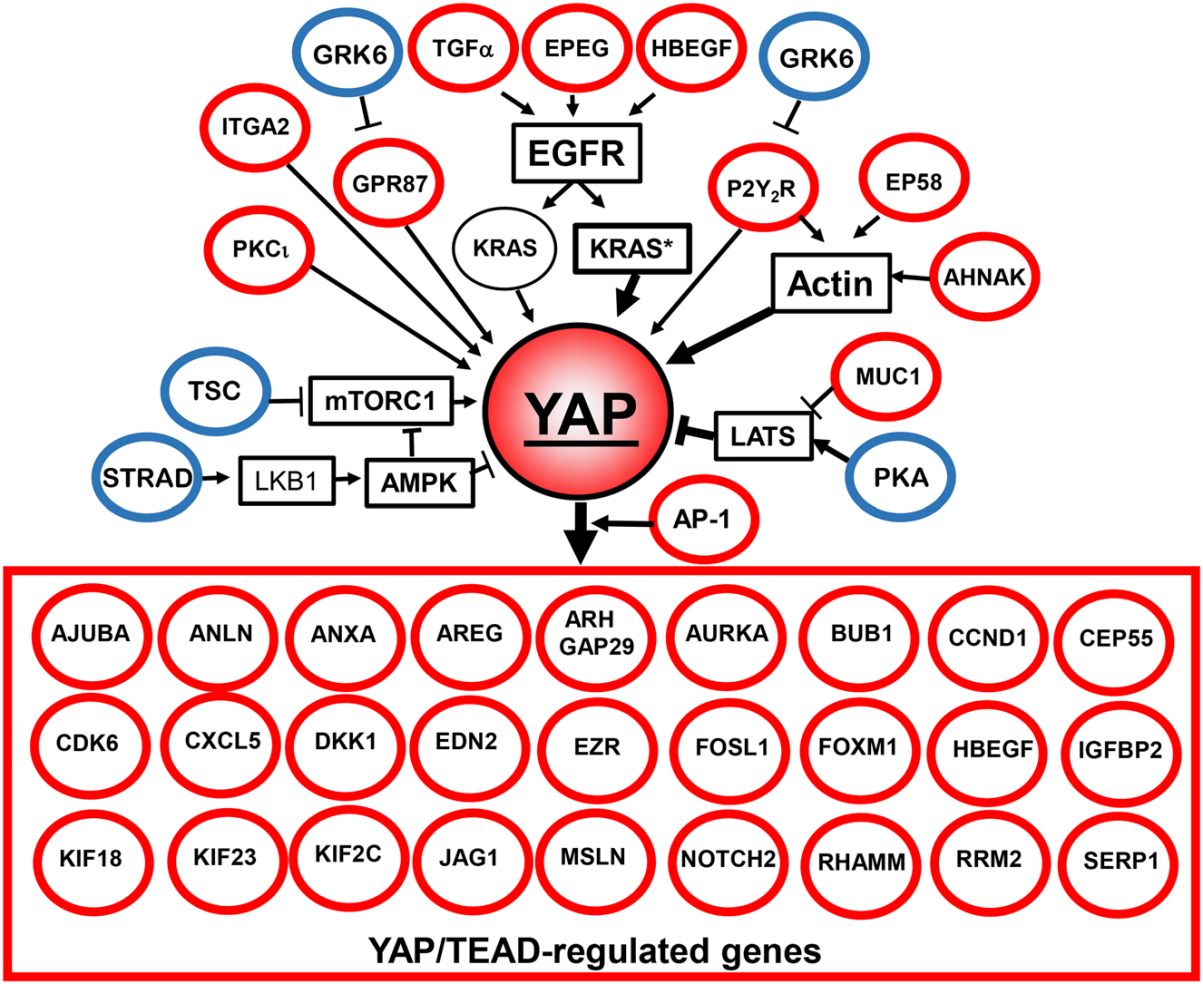
YAP signaling is associated with unfavorable prognosis for PDAC. Multiple YAP/TEAD-regulated genes are associated with unfavorable survival of PDAC patients (indicated in red). Growth factors (TGFα, EPEG, and HBEGF), a specific integrin (*ITGA2*), GPCRs (*P2Y*_*2*_*R*, *GPR87*) or an inhibitor of the Hippo pathway (*MUC1*) that stimulate YAP activity are also associated with unfavorable survival in PDAC. Conversely, YAP inhibitory pathways, including STRAD/LKB-1, PKA/LATS, and TSC/mTORC1 are associated with a favorable prognosis (indicated in blue). The key feature is that each component of the network has an impact on survival of PDAC patients, as derived from the Pathology Atlas^49^, as well as additional references cited in the text. An unfavorable prognosis for PDAC is in red and a favorable prognosis for PDAC is in blue. All prognostic associations are highly statistically significant (*p* < 0.001). Further details are in the text

As indicated in previous sections, YAP and TAZ constitute points of convergence of multiple upstream pathways and in turn, regulate the expression of multiple genes. In most cases, the regulation of gene expression by YAP/TAZ/TEAD occurs via distal enhancer elements^50^. We performed a correlation analysis between mRNA expression of genes that are downstream targets of YAP/TAZ in a variety of cell types and survival of PDAC patients by mining the data that are available in the recently available Pathology Atlas^49^. As highlighted in Fig. 4, multiple YAP/TEAD-regulated genes are significantly associated (*p* < 0.001) with unfavorable prognosis in PDAC^49^. These include *AJUBA*^50^, *ANLN*^51^, *ANXA3*^50^, *AREG*^28^, *ARHGAP19*^52^, *ARHGAP29*^53^, *AURKA*^19^, *BUB1*^19, 50^, *CCNA2*^50^, *CCND1*^50, 54^, *CDK6*^50, 55^, *CEP55*^50^, *CXCL5*^31, 47, 50^, *DKK1*^50, 56^, *EDN2*^50, 57^, *EZR*^50, 58^, *FOXM1*^59^, *HBEGF*^50^, *IGFBP2*^60^, *JAG1*^50, 61^, *KIF2C*^50^, *KIF18B*^50^, *KIF23*^50^, *MSLN*^50, 62^, *NOTCH2*^63^, *PRMT5*^50^, *RRM2*^50^, *SERP1*^50^, *RHAMM*^23^, and *ZWILCH*^50^. Each reference identifies the study or studies that connected the expression of the corresponding gene to YAP/TAZ/TEAD activity. In addition, independent studies using different patient populations demonstrated that the expression of YAP/TAZ/TEAD-regulated genes, including *ANLN*^64^, *AREG*^65^, *CCND1*^66^, *DKK1*^67^, *EZR*^58^, *FOSL1*^68^, *FOXM1*^69^, *MSLN*^70^, *RHAMM*^71^, *RRM2*^72^, and *SERP1*^73^, is associated with shorter survival in patients with PDAC.

It is important to emphasize that the protein products of YAP/TEAD-regulated genes that are associated with PDAC survival regulate a set of fundamental biological processes in cancer development. For example, AREG, HBEGF, CCND1, and RRM2 stimulate cell proliferation; AURKA, BUB1, CEP55, KIF23, and ZWILCH participate in mitosis;^68, 74^ YAP cooperates with FOXM1 to promote chromosome instability^59^, and CXCL5 mediates communication between cancer cells and myeloid-derived suppressor cells^47^, which contributes to the immunosuppressive microenvironment characteristic of PDAC. Other YAP/TEAD-regulated gene products are involved in the regulation of developmental pathways, including NOTCH, DKK1/WNT, and the Hippo pathway itself via AJUBA^75^. AURKA also inhibits LKB1/AMPK signaling thereby leading to YAP activation (see below). Furthermore, proteins that are encoded by the YAP-regulated genes *EZR* and *IGFBP2* (ezrin and IGFBP2, respectively) promote metastasis and epithelial-to-mesenchymal transition (EMT) in pancreatic cancer cells^76^. In turn, EMT contributes to the loss of cell polarity and thus to inhibition of the Hippo pathway, thereby reinforcing YAP activation. It is also of interest that the expression of some YAP/TEAD-regulated genes, including *CTGF* (encoding for connective tissue growth factor) have not been identified as prognostic markers of PDAC, but are likely to play a role in pancreatic carcinogenesis^77^.

In addition to AREG (amphiregulin) and HBEGF (heparin-binding EGF), which stimulate YAP/TEAD signaling via autocrine/paracrine stimulation of EGFR^78^ as part of positive feedback loops, other EGFR ligands, including TGFα and epiregulin (EPEG) that stimulate YAP activity are also associated with shorter overall survival in PDAC (Fig. 4). Similarly, the increased expression of *MET*^79^, *ITGA2*^80^, *IQGAP1*^81^, *EZR*^82^, *MUC1*^83^, *PRKC*^84^, and *YES1*^85^ which enhance YAP co-transcriptional activity through different mechanisms, is associated with shorter survival in patients with PDAC (Fig. 4). Interestingly, the activator protein-1 (AP-1, dimer of JUN and FOS proteins) factors have been shown to potentiate YAP/TAZ/TEAD-dependent gene expression via enhancers rather than promoters^50^. In line with the notion that YAP/TAZ is at the center of a signaling network that is associated with patient survival, the expression of *FOSL1*, a component of AP-1, is also strongly associated (*p* < 0.001) with an unfavorable prognosis in PDAC, as has also been shown recently in an independent report^68^.

GPCR signaling is one of the major upstream signals that regulate YAP/TAZ activation in a variety of systems, including PDAC^31^. In this context, the expression of genes encoding the lysophosphatidic acid (LPA) receptor *GPR87*^86^, the purinergic GPCR *P2Y*_*2*_*R*^87^ and the GPCR agonist *EDN2* (endothelin 2)^57^, a downstream target of YAP, are also associated with an unfavorable prognosis in PDAC. A recent independent study confirmed that overexpression of *GPR87* is correlated with a poor prognosis in PDAC^88^. By contrast, the expression of GRK6, which phosphorylates GPCRs thus opposing their signaling output^89^, is associated with a favorable survival prognosis in PDAC (Fig. 4).

**Fig. 5:**
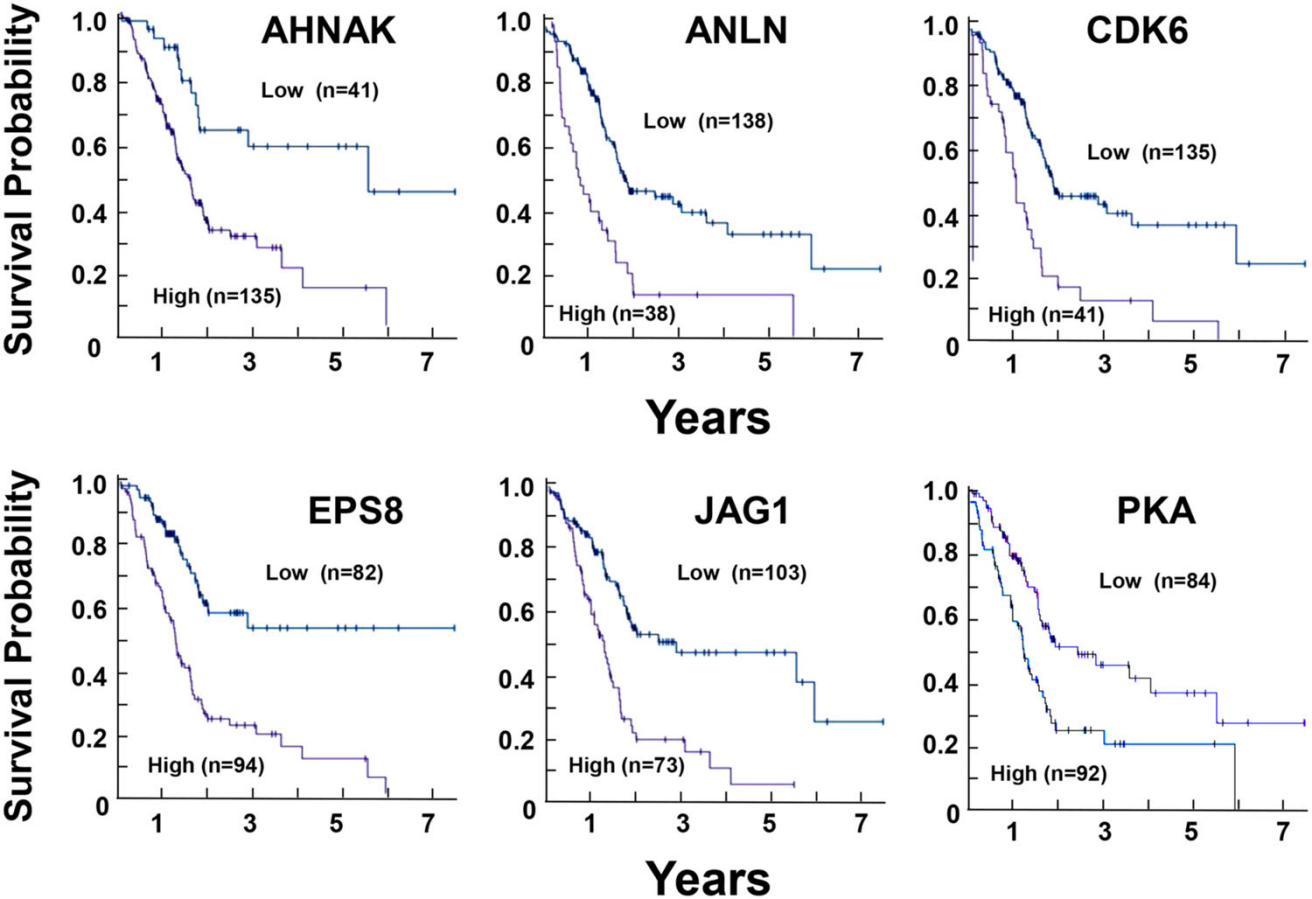
Kaplan–Meier plots for gene expression of the YAP signaling network in PDAC. Images were reproduced from the Human Protein Atlas (version 17) available at www.proteinatlas.org. The links to the specific genes shown are as follows: AHNAK, http://www.proteinatlas.org/ENSG00000124942AHNAK/pathology/tissue/pancreatic±cancer, ANLN, http://www.proteinatlas.org/ENSG00000011426ANLN/pathology/tissue/pancreatic±cancer CDK6, http://www.proteinatlas.org/ENSG00000105810-CDK6/pathology/tissue/pancreatic±cancer EPS8, http://www.proteinatlas.org/ENSG00000151491-EPS8/pathology/tissue/pancreatic±cancer JAG1, http://www.proteinatlas.org/ENSG00000101384-JAG1/pathology/tissue/pancreatic±cancer PKA, http://www.proteinatlas.org/ENSG00000072062-PRKACA/pathology/tissue/pancreatic±cancer

As mentioned above, the organization of the actin cytoskeleton is a master regulator of YAP/TAZ nuclear localization and activity^90, 91^. Interestingly, a number of proteins that are encoded by YAP/TEAD-regulated genes also influence the organization of the actin cytoskeleton, suggesting the existence of feedbacks loops. The epidermal growth factor receptor pathway substrate 8 (*Eps8*), a regulator of actin remodeling that is up-regulated in >70% of PDACs and induces YAP translocation to the nucleus and transcriptional activation^92^, is associated with an unfavorable prognosis in PDAC^49^. ANLN and AHNAK, proteins that are implicated in actin cytoskeleton organization^93^ and thus, potentially in YAP regulation, are also associated with unfavorable survival in PDAC. Indeed, *AHNAK*, *ANLN*, *CDK6*, *EPS8*, and *JAG1* are among the genes with the highest significance associated with unfavorable prognoses in PDAC (Kaplan–Meier plots in Fig. 5; *p* < 4.1e^−6^) and are all either upstream or downstream of YAP/TEAD (Fig. 4).

## Pathways that oppose yap signaling are favorable prognostic markers in pdac

In recent years, it has become apparent that, in addition to the cascade of Hippo kinases, other important pathways also inhibit YAP/TAZ functions. AMP–activated protein kinase (AMPK) is a well-known sensor of cellular energy that is activated when ATP concentrations decrease and 5′-AMP concentrations increase^94^. AMPK opposes YAP activity at different levels, including direct phosphorylation of YAP at Ser-94, a key residue for the interaction between YAP and TEAD^35, 95^. The tumor suppressor LKB-1/STK11 is the major kinase for phosphorylating the AMPK activation loop at Thr-172^96^. Interestingly, STE20-related adaptor (STRAD), a co-factor that allosterically stimulates LKB-1 activity and thus promotes AMPK activity, is a favorable prognostic marker in pancreatic cancer (Fig. 4). Furthermore, the protein kinases of the MARK family (e.g., MARK1) that also function downstream of LKB-1 and repress YAP activity^97^ are also associated with a favorable prognosis in pancreatic cancer.

A number of other studies have indicated that the cAMP/PKA pathway also inhibits YAP activity^98^, at least in part via stimulation of LATS kinases^99^. Accordingly, expression of PKA is associated with a favorable prognosis in PDAC, presumably via inhibition of YAP function (Fig. 4).

As mentioned above, mTORC1 is part of an amplification loop that leads to YAP expression and enhanced activity. The heterodimer of the tumor suppressors TSC2 (tuberous sclerosis 2, also known as tuberin) and TSC1 (tuberous sclerosis 1, also known as hamartin) opposes mTORC1 signaling^100, 101^ by acting as a GTPase-activator protein for the small G protein Rheb, a potent activator of mTORC1 signaling in its GTP-bound state^102, 103^. Importantly, increased expression of *TSC2* and *TSC1* is associated with a favorable prognosis for patients with PDAC (Fig. 4).

## Targeting the yap signal transduction pathway

As a result of the developments discussed in this article, there is intense interest in targeting YAP/TAZ in PDAC therapy and chemoprevention. Although inhibition of the activity of transcription factors or their co-activators is a challenging strategy, recent preclinical and epidemiological evidence suggest new approaches for targeting the YAP/TAZ signal transduction pathway.

Many studies have shown that the mevalonate pathway is markedly up-regulated in several epithelial cancers via mutant p53^95, 104, 105^ and Akt/mTORC1^95^. Statins are specific inhibitors of 3-hydroxy-methylglutaryl (HMG) CoA reductase^106^ which is the rate-limiting enzyme in the generation of mevalonate, the first step in the biosynthesis of isoprenoids leading to farnesyl pyrophosphate (FPP), geranylgeranyl pyrophosphate (GG-PP) and cholesterol. Transfer of the geranylgeranyl moiety to a COOH-terminal cysteine of Rho GTPases is critical for their function in signal transduction. In turn, active Rho (i.e., Rho-GTP) is essential for YAP/TAZ activation via actin cytoskeletal organization. Statins, which are usually well tolerated and generally safe, are used to treat hypercholesterolemia and prevent cardiovascular diseases. Although initially inconsistent, mounting epidemiological studies indicate that the use of statins is associated with a reduced risk and beneficial effects in PDAC^107–111^, especially in men^110, 111^. For example, a recent large study demonstrated that statin use was associated with a 34% reduced risk of PDAC, with a stronger association in male subjects^110^. In addition to their potential use in primary prevention, statins have also been shown to improve the survival of patients after resection of primary PDAC tumors, indicating a possible role of statins in the prevention of secondary PDAC^107, 108, 112^. Of great interest, a high-throughput screen of compounds capable of altering the subcellular localization of YAP led to the identification of statins as potential YAP inhibitors via the inhibition of Rho^113^. An independent study that examined the regulation of the expression of the receptor for hyaluronan-mediated motility (RHAMM) also led to the discovery that statins inhibit YAP/TAZ activity^23^. Consequently, statins provide a plausible strategy for targeting YAP/TAZ function and thus, an explanation for the mechanism by which these drugs appear to exert beneficial effects in PDAC. In view of these considerations, prospective clinical trials targeting YAP/TAZ with statins in primary or secondary PDAC chemoprevention are needed.

Metformin (1,1-dimethylbiguanide hydrochloride) is the most widely prescribed drug for the treatment of type 2 diabetes mellitus (T2DM) worldwide^94, 114^. Epidemiological studies suggest that administration of metformin may reduce cancer incidence and mortality in diabetic patients^115^. A recent meta-analysis indicated that metformin improved survival in PDAC patients with resection and patients with locally advanced tumors, but not in patients with metastatic PDAC^116^. In mechanistic studies, we demonstrated that metformin potently stimulated AMPK activation in intact human PDAC cells that were cultured with physiological concentrations of glucose in the medium^117, 118^, and inhibited mTORC1, ERK and mitogenic signaling via AMPK at low concentrations^117–119^. Metformin also inhibited the growth of PDAC xenografts^29, 120^ and the development of PanINs and PDAC in KC mice^121^. As indicated above, AMPK, a well-known sensor of cellular energy^94^, opposes YAP function via direct phosphorylation of YAP at Ser-94^122, 123^ as well as by phosphorylation of HMG-CoA reductase at Ser-872, which inhibits mevalonic acid synthesis^124^. These studies imply a connection between cellular energy status, lipid metabolism, AMPK and YAP/TAZ function. Because statins and metformin interfere with YAP function through different mechanisms, it is plausible that a combination of these agents additively or synergistically may suppress YAP/TAZ activity and thereby exerts cancer-preventive activity at low concentrations of each agent.

As indicated in Fig. 4, YAP/TAZ leads to an increase of the Notch pathway, which is likely to contribute to the oncogenic effects of YAP/TAZ. Indeed, pharmacological inhibitors of Notch activation reduced PDAC xenograft growth in preclinical models^125^ but were not effective in PDAC patients with advanced disease^126^. Upstream of YAP, inhibitors of EGFR have already shown a small favorable effect in PDAC^127^, however, other pathways can bypass EGFR. mTORC1 is part of an amplification loop that leads to YAP expression and enhanced activity, and the heterodimer of TSC2 and TSC1 which opposes mTORC1 signaling^100, 101^, is associated with a favorable prognosis for patients with PDAC. A number of mTOR inhibitors are available but their efficacy decreases with time and YAP provides one route for escape^128^. There is a clear need for further studies using combinations of drugs that target the YAP network at different levels to determine their possible anticancer activity.

A number of small molecule tyrosine kinase inhibitors, including dasatinib and pazopanib, induce YAP/TAZ phosphorylation and cytoplasmic degradation and reduce their nuclear translocation, suggesting another approach for restraining YAP/TAZ activity^129^. Although these inhibitors frequently induce drug resistance via enhancement of compensatory pathways^130^ these tyrosine kinase inhibitors could be considered as part of a combinatorial strategy.

A number of laboratories have searched for compounds that inhibit the nuclear localization of YAP and/or the interaction of YAP with TEAD^131^. Verteporfin (trade name Visudyne), a member of the porphyrin family, is used in photodynamic therapy of ophthalmological diseases to destroy new abnormal vessels with few side effects. Verteporfin, without light activation, has been reported to inhibit YAP/TEAD complex formation, thereby acting as a suppressor of cell growth^132–134^. However, in addition to its putative inhibitory effect on YAP/TEAD function, verteporfin also induces protein cross-linking via a non-enzymatic mechanism^135, 136^. Thus, verteporfin provides a plausible strategy for interfering with YAP/TEAD function within cells, but it has additional cellular effects and therefore its specificity as a YAP/TEAD antagonist has been questioned.

Mammalian Vestigial-like 4 (VGLL4) is a tumor suppressor that does not bind directly to DNA but competes with YAP for binding TEADs^137, 138^. A peptide capable of mimicking the function of VGLL4 suppressed tumor growth *in vitro* and *in vivo*, implying that disrupting the YAP-TEAD interaction by a VGLL4-mimicking peptide may be a therapeutic strategy for inhibiting YAP-mediated cell proliferation^137^. However, this approach has limitations, including the permeability of the peptide across the plasma membrane and its stability. Importantly, crystal structures of YAP/TEAD^139^ and TAZ/TEAD^140^ complexes have been solved, opening new avenues for computational modeling of compounds that disrupt these molecular complexes within intact cells^141, 142^.

## Conclusion and implications

PDAC is generally a fatal disease with no efficacious treatment modalities. It is important that strategies to prevent the disease be explored, especially since its incidence is projected to increase markedly in the next decade. YAP and TAZ, the major downstream targets of the Hippo tumor suppressor pathway (Fig. 1), are also regulated by a multitude of other inputs, including KRAS (Fig. 2). The data discussed in this article indicate that not only YAP (Fig. 3) but also many components of the YAP network are associated with overall survival in PDAC (Fig. 4). Indeed, multiple downstream targets of YAP/TAZ/TEAD are associated with unfavorable survival of PDAC patients. Similarly, multiple components that stimulate YAP activity, including AP-1 which synergizes with YAP (*FOSL1*) and growth factors (TGFα, EPEG, and HBEGF), GPCRs (*P2Y*_*2*_*R*, *GPR87*), a specific integrin (*ITGA2*) or an inhibitor of the Hippo pathway (*MUC1*), are all convincingly associated with unfavorable survival in PDAC (Fig. 4). In sharp contrast, increased expression of YAP inhibitory pathways (LKB-1, PKA, TSC) portends a favorable prognosis. All of these associations which have been derived from the recently published interactive open-access database (www.proteinatlas.org/pathology) that allows genome-wide exploration of the impact of individual proteins on survival outcomes, emphasize that increased expression of the YAP signaling web correlates with poorer survival of patients with PDAC. This analysis convincingly supports the notion that the YAP network is a target for therapeutic and preventive strategies in PDAC. The association of patient survival to the YAP/TAZ signaling network strongly suggests a need for novel combinatorial strategies for targeting the YAP network in PDAC, one of the most lethal diseases.

Upstream of YAP, EGFR inhibitors have already shown a small favorable effect in PDAC^127^, however, other pathways, including other tyrosine kinase receptors, can bypass EGFR and thus stimulate YAP leading to drug resistance. As indicated above, statins and metformin interfere with YAP function through different mechanisms, and recent epidemiological studies indicate that their administration is associated with beneficial effects in PDAC^143, 144^. Downstream of YAP, inhibitors of NOTCH pathway activation have shown activity in preclinical models, but were not pursued further because they did not exert any response in advanced PDAC patients. Thus, there are drugs, some of which are FDA-approved and in clinical use, that target the YAP network at different levels, thus providing a rationale for designing combinatorial strategies in both preclinical settings and in future clinical trials for PDAC. Furthermore, there are major efforts to develop potent compounds that disrupt YAP/TEAD and TAZ/TEAD molecular complexes within intact cancer cells.

Given the strong correlation of the YAP signaling network with patient survival that is demonstrated in this article, it is conceivable that a combination of these drugs will suppress YAP/TAZ activity synergistically and thereby exert cancer-preventive activity at low concentrations of each agent, a proposition that warrants further experimental, preclinical and clinical work.
